# Supplementary material for: Direct oxygen isotope effect identifies the rate-determining step of electrocatalytic OER at an oxidic surface
Source: Nat Commun. 2018 Nov 1;9:4565. doi: 10.1038/s41467-018-07031-1 (PMC6212532; doi:10.1038/s41467-018-07031-1)
Supplement: Supplementary file 1 — Supplementary Information [file 41467_2018_7031_MOESM1_ESM.pdf]

Electronic Supplementary Information to:

Direct oxygen isotope effect identifies the rate-determining step of electrocatalytic OER at an oxidic surface

Haschke *et al.*

Supplementary Figures

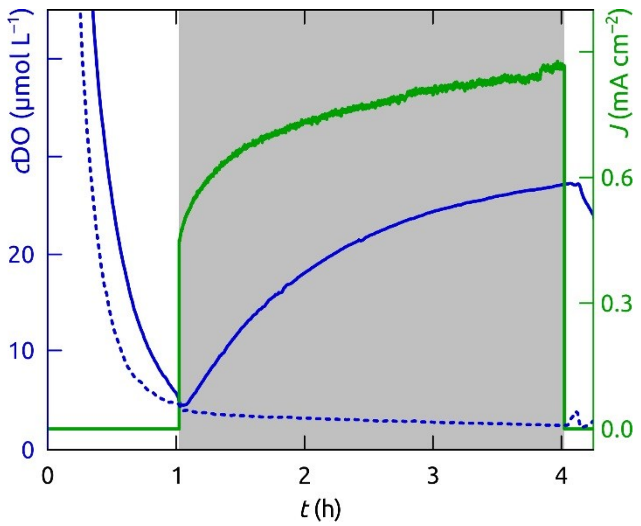

Supplementary Figure 1. Time-dependent oxygen evolution of Fe_2O_3 electrodes. Any contributions of contamination by atmospheric oxygen due to improper sealing could be excluded via a control experiment, in which the nanoporous Fe_2O_3 electrode was left in open circuit (zero current, blue dashed line). Furthermore, the successful evolution of dioxygen upon steady-state electrolysis was then directly quantified with an optical oxygen sensor (optode). The figure exemplarily shows the time-dependent increase in the dissolved oxygen (DO) concentration and the corresponding current density J for an applied potential E of +1.30 V vs. Ag/AgCl (overpotential η = 0.68 V). The Fe_2O_3 electrode is exposed to a pH 7 aqueous KH_2PO_4 electrolyte. The grey area represents the duration of steady-state electrolysis (η = 0.68 V), whereas the system is left in open circuit during the preliminary degassing procedure. J is calculated from the experimental current intensity I and the macroscopically defined sample area A.

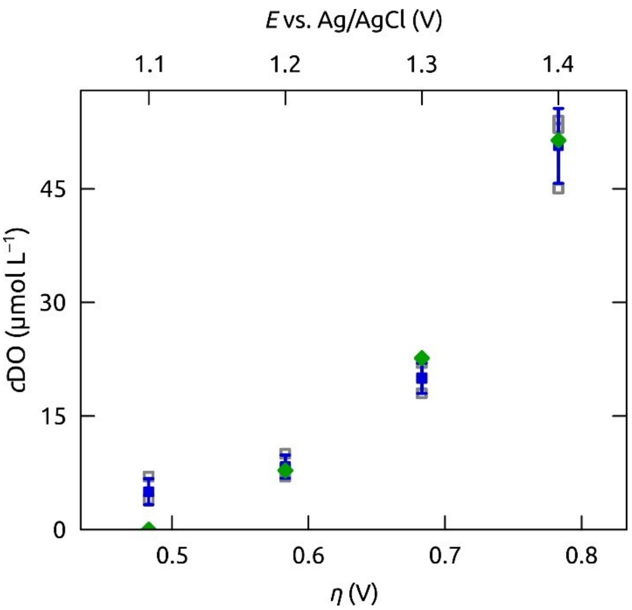

Supplementary Figure 2. Comparative quantification of DO concentrations. The suitability of the isotope ratio mass spectrometry (IRMS) method for our system is proven by a quantitative comparison of DO concentrations c_{DO} measured with IRMS and optical sensing for steady-state electrolysis (3 h) at nanoporous Fe_2O_3 electrodes. IRMS yielded mean DO concentrations of 5 µmol L^{-1} (for $\eta = 0.48$ V) to 50 µmol L^{-1} (for $\eta = 0.78$ V), which are in agreement with the optode measurements. The exponential increase in DO coincides with the J trend (see also **Supplementary Fig. 3**).The electrolysis experiments are performed at various applied overpotentials $0.48 \leq \eta \leq 0.78$ V (potentials E vs. Ag/AgCl: $+1.10 \leq E \leq +1.40$ V) in a pH 7 aqueous KH_2PO_4 electrolyte. For IRMS, concentrations of DO are presented for three nominally identical individual samples (grey empty squares) and the corresponding average values with standard deviations (blue squares and bars), whereas each value plotted for the optical oxygen sensor (green rhombuses) represents a Δc_{DO} between measurements performed before and after electrolysis.

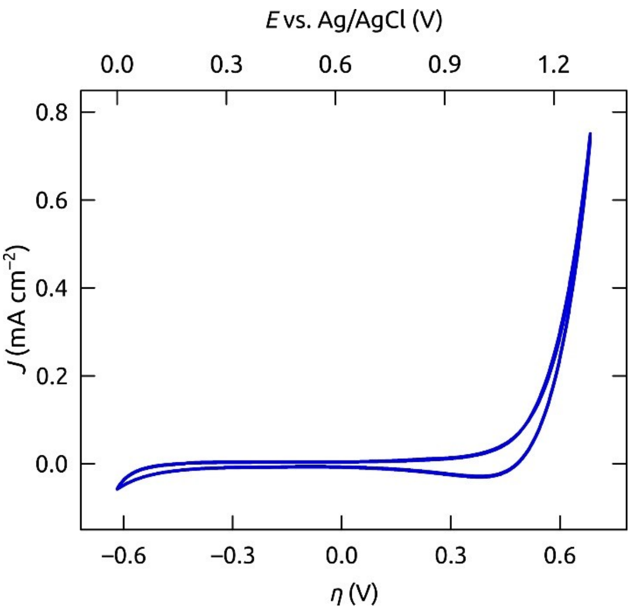

Supplementary Figure 3. Current-voltage characteristics of nanoporous Fe_2O_3 electrodes. In accordance with the Butler-Volmer equation,[1-5] the electrocatalytic current density J shows the typical exponential dependence on applied overpotential η. This trend is in agreement with the measured DO concentrations (see **Supplementary Fig. 2**). The cyclic voltammogramm is recorded vs. an Ag/AgCl electrode in a pH 7 aqueous KH_2PO_4 electrolyte (scan rate: 50 mV s^{-1}).

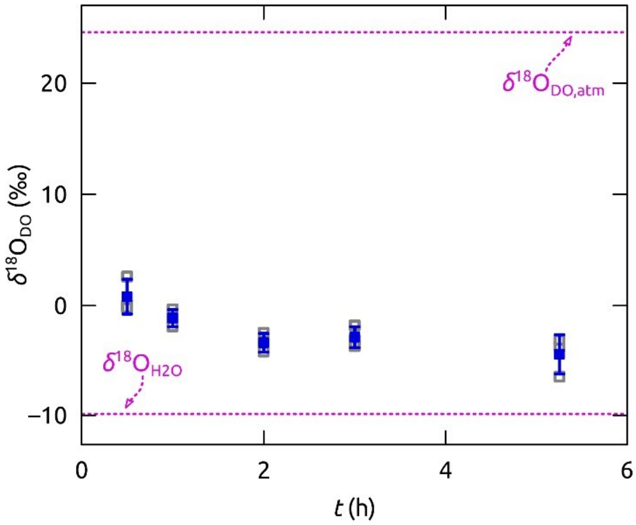

Supplementary Figure 4. Time-dependent $\delta^{18}O_{DO}$ composition of O_2 evolved at nano-porous Fe_2O_3 electrodes. As an additional control experiment to exclude that negligible adventitious atmospheric O_2 influences the results, we compare $\delta^{18}O_{DO}$ values for different electrolysis durations. The duration has negligible influence on the isotopic composition within a maximum error margin of 5‰. For comparison, our discussion on $\delta^{18}O_{DO}$ for various applied overpotentials is based on variations larger than 30‰ (**Figure 2** in the main manuscript text). The DO compositions of $\delta^{18}O_{DO}$ evolved upon steady-state electrolysis are studied in a pH 7 aqueous KH_2PO_4 electrolyte for different electrolysis durations $0.50 \leq t \leq 5.25$ h (constant applied overpotential $\eta = 0.68$ V). Values of $\delta^{18}O_{DO}$ are presented for three nominally identical individual samples (grey empty squares) and the corresponding average values with standard deviations (blue squares and bars). Isotopic compositions measured for atmospheric O_2 dissolved in the aqueous electrolyte and electrolyte H_2O are given as control values (dashed lines): $\delta^{18}O_{DO,atm} = +24.6‰$, $\delta^{18}O_{H2O} = −9.8‰$.

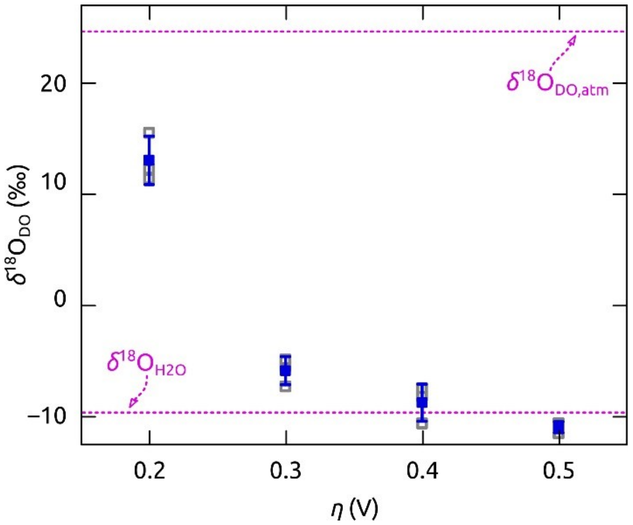

Supplementary Figure 5. Potential-dependent $\delta^{18}O_{DO}$ composition of O_2 evolved at nanoporous Ir electrodes. The use of a completely different electrocatalyst, namely, oxide-covered iridium, shows the same trend of $\delta^{18}O_{DO}$ decline with overpotential η increase as observed for the Fe_2O_3 electrode. Due to the significantly higher catalytic activity of Ir, steady-state electrolyses were conducted at lower η. The compositions of $\delta^{18}O_{DO}$ evolved upon steady-state electrolysis at Ir electrodes are studied in a 0.1 M H_2SO_4 electrolyte between $0.20 \leq \eta \leq 0.50$ V (potentials E vs. Ag/AgCl: $+1.20 \leq E \leq +1.50$ V, constant electrolysis duration $t = 0.5$ h). Values of $\delta^{18}O_{DO}$ are presented for three nominally identical individual samples (grey empty squares) and the corresponding average values with standard deviations (blue squares and bars). Isotopic compositions measured for atmospheric O_2 dissolved in the aqueous electrolyte and electrolyte H_2O are given as control values (dashed lines): $\delta^{18}O_{DO,atm}$ = +24.7‰, $\delta^{18}O_{H2O}$ = −9.6‰.

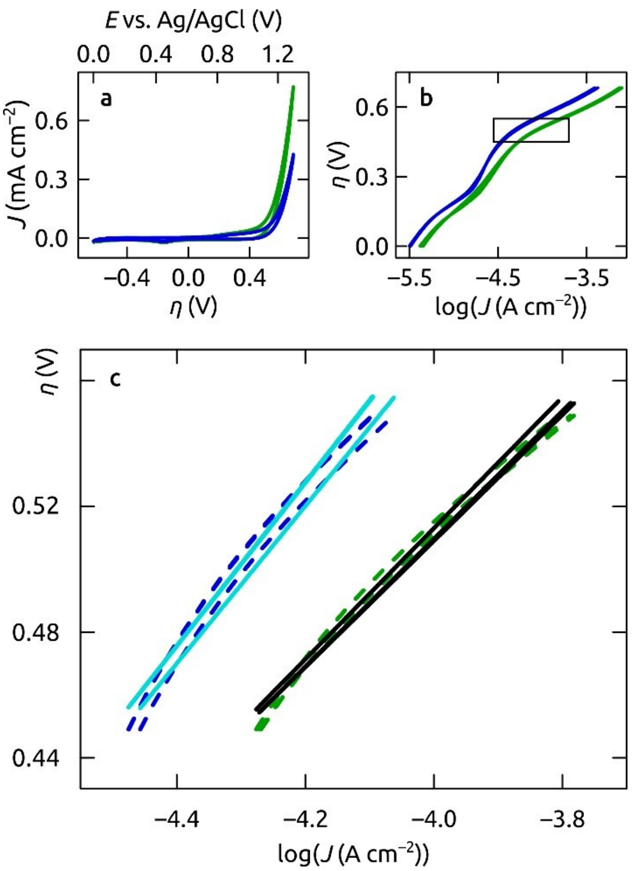

Supplementary Figure 6. Kinetic deuterium isotope effects for the OER at nanoporous Fe_2O_3 electrodes. For the determination of the kinetic deuterium isotope effect, the current-potential behaviour is studied for nanoporous Fe_2O_3 electrodes in protio (green curves) and deuterio (blue curves) 0.1 M KH_2PO_4 solutions (pH 7) under an inert N_2 atmosphere. **a** In cyclic voltammetry, nanoporous Fe_2O_3 electrodes reveal constant current-potential curves for four consecutive cycles in both electrolytes (scan rate: 5 mV s^{-1}). The use of deuterated water significantly shifts the OER onset and decreases its activity. **b** The corresponding Tafel plots are presented for the OER region ($0.00 \leq \eta \leq 0.78$ V, three individual measurements each). **c** Tafel plots of the OER onset region ($0.45 \leq \eta \leq 0.55$ V) obtained for protio (dashed blue lines) and deuterio (dashed green lines) solutions with the corresponding linear fits (cyan and black lines for protio and deuterio, respectively; $R^2 \geq 0.988$). The ^{2}H KIE is determined from the Tafel plots in the OER onset region according to the method published by Tse et al.[6] (where the reaction is not significantly influenced by mass transport[5]). This method provides the exchange current densities for the OER in H_2O and ^2H_2O (J_0^H and J_0^D), which are directly proportional to the standard rate constants k_0,[5] so that the ratio J_0^H/J_0^D is equal to the ^{2}H KIE, k_0^H/k_0^D (see also **Supplementary Table 2**).

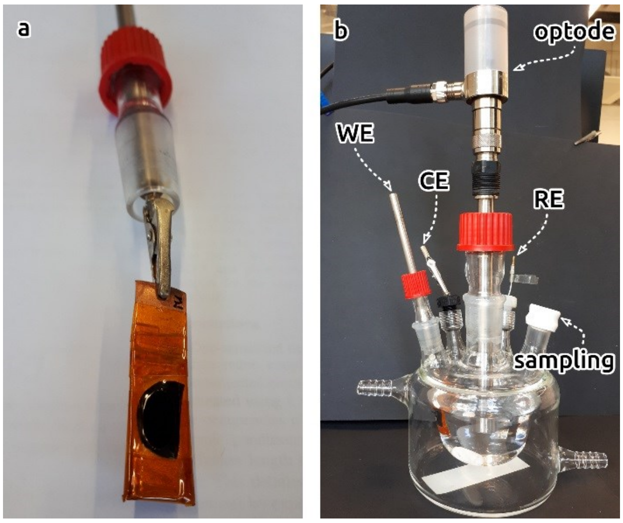

Supplementary Figure 7. Photographs of the measurement setup. **a** Nanoporous Fe_2O_3 working electrode of defined surface area and **b** the electrochemical measurement setup. A more detailed description can be found in the Methods section in the main manuscript text.

Supplementary Tables

Supplementary Table 1. Summary of the potential effect of residual adventitious DO on the experimentally determined $\delta^{18}O_{DO}$ values. $\delta^{18}O_{exp}$ represents the isotopic composition calculated for the experimentally determined (uncorrected) DO concentration. $\delta^{18}O_{corr}$ corresponds to the isotopic compositions of DO corrected for O_2 contamination. $\delta^{18}O_{exp}$ and $\delta^{18}O_{corr}$ are presented for the different applied overpotentials η. See **Supplementary Note 2** for more information.

η / V	$\delta^{18}O_{exp}$ / ‰	$\delta^{18}O_{corr}$ / ‰	error / ‰
0.48	21.0	20.2	0.8
0.58	9.7	7.8	1.9
0.68	−2.8	−4.1	1.3
0.78	−9.6	−10.2	0.6

Supplementary Table 2. Summary of the Tafel data determined from linear regressions of the Tafel plots. The Tafel plots were measured in protio and deuterio electrolytes for nanoporous Fe_2O_3 (**Supplementary Fig. 6**). The Tafel slopes and intercepts are given as mean values from three nominally identical individual measurements. Linear regression yields Tafel slopes b = 204 mV for H_2O and 257 mV for ^2H_2O. These values are in line with the lower OER activity observed in deuterated water (**Supplemenatry Fig. 6a**). Both Tafel slopes are not in the theoretically expected range of 40 to 120 mV dec^{-1},[7] which can be related to the nanoporous structure of our electrodes.[8,9] The slopes and intercepts are used to calculate the corresponding exchange current densities J_0 and the kinetic deuterium isotope effect ^{2}H KIE = k_H/k_D. Within the presented η-region, the calculated ^{2}H KIE is 0.56. Varying the η range for the fit yields KIE values between 0.56 and 0.96 (these are always inverse).

	Tafel slope / mV	intercept / V	J_0 / µA cm^{-2}	^{2}H KIE
H_2O	204	1.33	3.18×10^{-7}	0.56
D_2O	257	1.61	5.72×10^{-7}	

Supplementary Notes

Supplementary Note 1. Using Faraday's law,[10,11] we can compare the experimentally measured amount of dissolved dioxygen *n*(DO) = 2.6 µmol (η = 0.68 V, **Supplementary Fig. 2**) with the theoretical total amount of O_2 produced in the OER, *n*(O_2(total)). According to the total charge *q* passed during 3 h of electrolysis (**Supplementary Fig. 1**), *n*(O_2(total)) amounts to 8.5 µmol. Thus, approximately 6 µmol O_2 (two thirds of the total amount of O_2 produced) are distributed in the gas phase. This value is lower than the thermodynamic equilibrium value *n*($O_2(g_{Henry})$) of 44 µmol calculated from Henry's law (assuming $H_{cp}(O_2) = 1.3 \times 10^{-5}$ mol m^{-3} Pa^{-1}), that is, the theoretical amount of oxygen present in the gas phase at equilibrium.[12] The lower value determined experimentally is related to the slow exchange at the gas / water interface. Independent of the distribution of O_2 in the liquid and gas phase, these preliminary data demonstrate the applicability of direct IRMS analysis on O_2 in small DO quantities, and thus, its suitability as a tool for the investigation of the OER kinetics in natural-abundance water.

Supplementary Note 2. Error calculation of baseline O_2 concentration effect. **Supplementary Fig. 1** indicates a non-zero DO concentration baseline after degassing the electrolyte and before the start of the electrolysis. This concentration of 3 µmol L^{-1} or 0.1 ppm corresponds to the standard Schlenk technique contamination level, as well as to the measurement limits of both the optrode and the mass spectrometric determination. Let us calculate which error this contamination might possibly cause. We base the calculation on a mass balance,

$$\delta^{18}\mathrm{O}_{\mathrm{exp}} = \frac{(\delta^{18}\mathrm{O}_{\mathrm{corr}} \cdot c_{\mathrm{corr}}) + (\delta^{18}\mathrm{O}_{\mathrm{bl}} \cdot c_{\mathrm{bl}})}{c_{\mathrm{exp}}} \quad (1)$$

with the following abbreviations:

- all $\delta^{18}O$ values refer to dissolved oxygen (DO);
- c_{exp} is the experimentally determined (uncorrected) DO concentration;
- c_{bl} is the concentration of the baseline O_2 concentration;
- c_{corr} represents the DO concentration corrected for O_2 contamination;
- $\delta^{18}O_{exp}$, $\delta^{18}O_{bl}$, $\delta^{18}O_{corr}$ represent the corresponding isotopic compositions.

Using

$$c_{\mathrm{corr}} = c_{\mathrm{exp}} - c_{\mathrm{bl}} \quad (2)$$

one obtains

$$\delta^{18}\mathrm{O}_{\mathrm{exp}} = \frac{[\delta^{18}\mathrm{O}_{\mathrm{corr}} \cdot (c_{\mathrm{exp}} - c_{\mathrm{bl}})] + (\delta^{18}\mathrm{O}_{\mathrm{bl}} \cdot c_{\mathrm{bl}})}{c_{\mathrm{exp}}} \quad (3)$$

Solving for $\delta^{18}O_{corr}$ yields

$$\delta^{18}\mathrm{O}_{\mathrm{corr}} = \frac{(\delta^{18}\mathrm{O}_{\mathrm{exp}} \cdot c_{\mathrm{exp}}) - (\delta^{18}\mathrm{O}_{\mathrm{bl}} \cdot c_{\mathrm{bl}})}{c_{\mathrm{exp}} - c_{\mathrm{bl}}} \quad (4)$$

As an example, let us calculate from equation 4 the corrected value at η = 0.58 V (the case in which the correction causes the largest change). We go from the assumption that the originally dissolved adventitious O_2 has equilibrated with the gas phase over the electrolysis duration, yielding $\delta^{18}O_{bl}$ = +24.6‰ and c_{bl} = 0.93 µmol L^{-1} (see page S3):

$$\frac{(9.7\,‰ \cdot 8.3\,\mu\mathrm{mol\,L^{-1}}) - (24.6\,‰ \cdot 0.9\,\mu\mathrm{mol\,L^{-1}})}{(8.3 - 0.9)\ \mu\mathrm{mol\,L^{-1}}} = 7.8\,‰ \quad (5)$$

This value deviates from the experimental one (9.7‰) by 1.9‰ — a deviation that is significant but does not affect the trend or the qualitative interpretation. This deviation is also within the drift determined experimentally (**Supplementary Fig. 4**). **Supplementary Table 1** summarizes the results obtained at the various overpotentials.

Supplementary References

1. Butler, J. A. V. Studies in heterogeneous equilibria. Part II.-The kinetic interpretation of the nernst theory of electromotive force. *Trans. Faraday Soc.* **19**, 729-733 (1924).
2. Butler, J. A. V. Studies in heterogeneous equilibria. Part III. A kinetic theory of reversible oxidation potentials at inert electrodes. *Trans. Faraday Soc.* **19**, 734-739 (1924).
3. Erdey-Gruz, T., Volmer, M. The theory of hydrogen overvoltage. *Z. Physik. Chem.* **150**, 203-213 (1930).
4. Erdey-Gruz, T., Volmer, M. Overvoltage of metals. *Z. Physik. Chem.* **A157**, 165-181 (1931).
5. Bard, A. J., Faulkner, L. R. *Kinetics of Electrode Reactions*. In: *Electrochemical Methods: Fundamentals and Applications.* (John Wiley & Sons, Inc., New York, 2001).
6. Tse, E. C. M., Hoang, T. T. H., Varnell, J. A., Gewirth, A. A. Observation of an inverse kinetic isotope effect in oxygen evolution electrochemistry. *ACS Catalysis.* **6**, 5706-5714 (2016).
7. Doyle, R. L., Lyons, M. E. G. *The Oxygen Evolution Reaction: Mechanistic Concepts and Catalyst Design*. In: *Photoelectrochemical Solar Fuel Production: From Basic Principles to Advanced Devices* (Springer International Publishing AG, Cham, 2016).
8. Gemmer, J., Hinrichsen, Y., Abel, A., Bachmann, J. Systematic catalytic current enhancement for the oxidation of water at nanostructured iron(III) oxide electrodes. *J. Catal.* **290**, 220-224 (2012).
9. Schlicht, S., Haschke, S., Mikhailovskii, V., Manshina, A., Bachmann, J. Highly reversible water oxidation at ordered nanoporous iridium electrodes based on an original atomic layer deposition. *ChemElectroChem.* **5**, 1259-1264 (2018).
10. Faraday, M. Experimental researches in electricity. – Seventh series. *Phil. Trans. Roy. Soc.* **124**, 77-122 (1934).
11. Jensen, W. B. Faraday’s laws or Faraday’s law? *J. Chem. Educ.* **89**, 1208-1209 (2012).
12. Sander, R. Compilation of Henry's law constants (version 4.0) for water as solvent. *Atmos. Chem. Phys.* **15**, 4399-4981 (2015).
